# Association between relative muscle strength and cardiovascular disease among middle-aged and older adults in China

**DOI:** 10.1186/s12889-024-19473-y

**Published:** 2024-07-18

**Authors:** Jin-jin Ji, Meng-jie Zhao, Meng-li Xiao, Hui-e Zhang, Qin Tan, Yu-rong Cheng, Fang Lu

**Affiliations:** 1 China Academy of Chinese Medicine Sciences, Xiyuan Hospital, Beijing, 100091 China; 2 NMPA Key Laboratory for Clinical Research and Evaluation of Traditional Chinese Medicine, Beijing, 100091 China; 3 National Clinical Research Center for Chinese Medicine Cardiology, Beijing, 100091 China

**Keywords:** Relative muscle strength, Cardiovascular disease, Muscle mass, Muscle strength

## Abstract

**Background:**

The association between sarcopenia and cardiovascular disease (CVD) is well known. However, the clinical diagnosis of sarcopenia is complex and not suitable for early clinical identification and prevention of CVD. Relative muscle strength (RMS) is a relatively quantitative and straightforward indicator, but its association with CVD remains unclear. Hence, the objective of this research was to investigate the correlation between RMS and CVD incidence.

**Methods:**

This was a cross-sectional study, using data from the China Health and Retirement Longitudinal Study (CHARLS) in 2011. CVD events were assessed through self-reported physician diagnoses. The RMS was determined by dividing the maximum grip strength by the appendicular skeletal muscle mass (ASM). This study used multivariate logistic regression and restricted cubic spline (RCS) curves to explore the correlation between RMS and CVD incidence. Additionally, we conducted subgroup analyses to provide additional evidence supporting the association between the two variables.

**Results:**

A total of 8,733 people were included in our study, with 1,152 (13.19%) CVD patients and 7,581 (86.81%) non-CVD patients. When the data were grouped according to quartiles (Q) of RMS, the inverse association between CVD and RMS remained statistically significant even after controlling for all potential confounding factors. Compared with participants in Q1 of RMS, the ORs (95% CIs) of CVD among those in Q2-Q4 were 0.99 (0.83, 1.17), 0.81 (0.67, 0.98), and 0.70 (0.57, 0.85), respectively. Moreover, the RCS results showed a negative linear correlation between the RMS and CVD incidence (P for nonlinearity = 0.555). Subgroup analysis revealed no significant interaction in any of the groups except for the sex group (P for interaction = 0.046).

**Conclusion:**

Our study indicated a stable negative correlation between RMS and CVD incidence. RMS is helpful for the early identification and prevention of CVD.

**Supplementary Information:**

The online version contains supplementary material available at 10.1186/s12889-024-19473-y.

## Background

Cardiovascular disease (CVD) is the main cause of death worldwide. The incidence of CVD has exhibited a remarkable surge, with an astounding 92.3% increase from 1990 to 2019, accompanied by a substantial increase in mortality rates, reaching 53.7% [1]. Moreover, the occurrence and frequency of CVD have increased significantly in recent years owing to the aging of the population [2]. CVD and its risk factors are increasingly severe challenges in developing countries or low-income countries, where the majority of cases are prevalent [3]. In China specifically, 40% of deaths can be attributed to CVD [4], placing an immense economic burden on affected families and healthcare systems [5]. Consequently, early identification and prevention of CVD are essential for middle-aged and elderly Chinese individuals.

Sarcopenia refers to the gradual decline in muscle strength, mass, and functionality over time [6, 7]. An increasing body of research indicates that sarcopenia has been linked to negative consequences, including falls, a decline in functionality, frailty, and death [8, 9]. The current widely accepted definition of sarcopenia was proposed by the European Working Group on Sarcopenia in Older People (EWGSOP) and endorsed by the Asian Working Group for Sarcopenia (AWGS) [10]. While physical function is increasingly utilized for assessing the severity of sarcopenia, a conclusive clinical diagnosis heavily relies on the essentiality of muscle strength and mass [6, 7].

To the best of our knowledge, extensive studies have consistently demonstrated a significant connection between sarcopenia and CVD [11, 12]. A study involving 11,863 people followed up for six years reported that sarcopenia was associated with a greater risk of CVD in middle-aged and elderly Chinese individuals [13]. Furthermore, it has been reported that sarcopenia often occurs before the onset of CVD and is a contributing factor to its progression [14]. However, the diagnostic process for sarcopenia defined by the AWGS is complex [15], which is detrimental to its clinical applicability. Relative muscle strength (RMS), which quantifies muscle strength in relation to muscle mass, has consistently shown a negative correlation with hypertension in previous studies even after accounting for variables that may influence the results [16]. Nevertheless, to our knowledge, no articles have explored the correlation between RMS and CVD incidence. Therefore, based on data from the China Health and Retirement Longitudinal Study (CHARLS), we explored the impact of RMS on CVD among middle-aged and elderly Chinese people while proposing a novel approach for the early identification and prevention of CVD.

## Methods

### Study population

This cross-sectional study used data from CHARLS, which aims to gather a comprehensive and high-quality dataset comprising micro-data that accurately represents Chinese individuals and households aged 45 years or older. The CHARLS national baseline survey was successfully conducted in 2011 and included interviews with a substantial sample size of 17,000 individuals residing in approximately 10,000 households across 150 counties and 450 villages. Subsequent surveys were conducted biennially in 2013, 2015, and 2018 and most recently in 2020. It is important to note that the ethical application for collecting human subject data within CHARLS has been duly approved by the Peking University Biomedical Ethics Review Committee; furthermore, all participants involved provided written informed consent. For further information regarding the CHARLS data collection procedures and outcomes, please refer to their official website (http://charls.pku.edu.cn/).

**Fig. 1:**
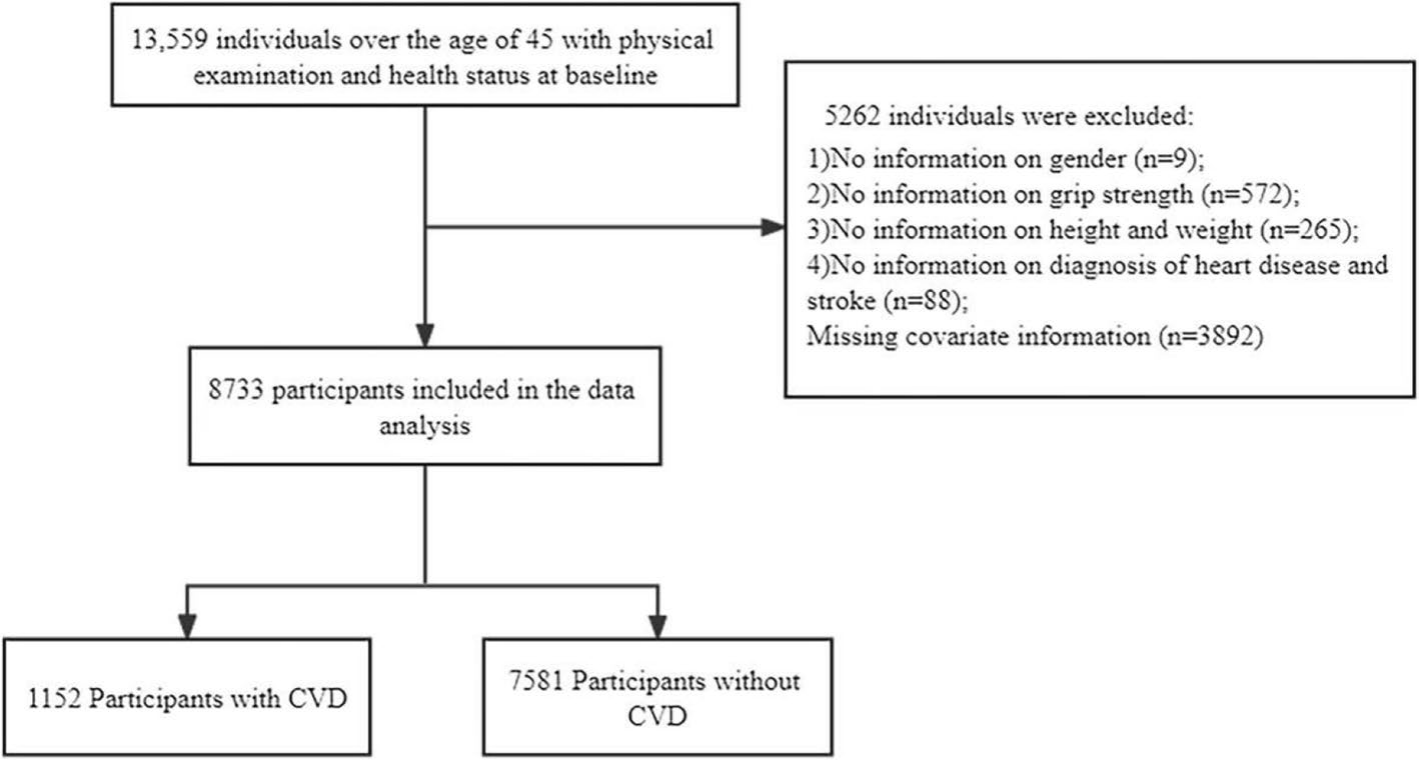
Flow chart of subject inclusion

The data utilized in our study were obtained from a baseline survey conducted in 2011, encompassing a substantial sample size of over 17,000 participants. Our inclusion criteria were 1) aged ≥ 45 years and 2) available physical examination records and physical health data (*n* = 13,559). Conversely, the exclusion criteria consisted of the following factors: 1) absence of sex information (*n* = 9); 2) lack of grip strength information (*n* = 572); 3) insufficient height and weight data (*n* = 265); 4) no history of heart disease or stroke (*n* = 88); and 5) inadequate covariate information available for analysis (*n* = 3,892), as illustrated in Fig. 1.

### Measurement of RMS

The RMS was defined as the grip strength divided by the ASM [16]. To assess grip strength, participants exerted maximum force on a dynamometer (YuejianTM WL-1000 dynamometer) as hard as possible, maintained the grip for a few seconds, and recorded the peak grip strength of both their dominant and non-dominant hands. Each hand was measured three times independently, with the highest mean value being registered.

ASM was obtained by the following formula: ASM = 0.193 * weight (kg) + 0.107 * height (cm) - 4.157 * sex—0.037 * age (years) - 2.631. For men, gender was set to 1; for women, it was set to 2. This equation accounts for ASM in adult individuals from China [17] and has been verified in previous studies [13, 18, 19].

### Assessment of CVD events

CVD events included myocardial infarction, coronary heart disease, angina, congestive heart failure, and stroke, which is consistent with previous research findings [20–22]. CVD events were assessed by self-reported physician diagnosis: "Has your doctor told you that you have been diagnosed with a heart attack, angina, coronary heart disease, heart failure, or other heart problems?" Alternatively, "Did the doctor tell you that you were diagnosed with a stroke?". Participants who responded affirmatively to these inquiries were classified as individuals with CVD.

### Covariates

The covariates collected at baseline included the following: 1) Sociodemographic characteristics included age, sex, level of education (elementary school or below; middle school; college or above), and marital status (married and other including separated, divorced, widowed and never married). 2) Physical data included systolic blood pressure (SBP), diastolic blood pressure (DBP), pulse, and BMI was calculated as weight (kg) divided by the square of height (m^2^). 3) Health-related factors included smoking status (Yes for smoking and No for never smoking), drinking status (Yes for drinking and No for never drinking), hypertension (participants with a self-reported physician-diagnosed history of hypertension or treated for hypertension, as well as those with a mean SBP ≥ 140 mmHg or a mean DBP ≥ 90 mmHg), diabetes (participants with a self-reported history of physician-diagnosed diabetes, taking glucose-lowering medications, or having a fasting blood glucose ≥ 7 mmol/L or glycosylated hemoglobin ≥ 6.5%). Other chronic diseases including dyslipidemia, kidney disease, and liver disease were self-reported physician diagnoses. 4) Laboratory test data included total cholesterol (TC), high-density lipoprotein cholesterol (HDL-C), low-density lipoprotein cholesterol (LDL-C), triglyceride (TG), C-reactive protein (CRP), and glycated haemoglobin (HbA1c) levels.

### Statistical analysis

The mean ± standard deviation (SD) was used to present quantitative data for variables that followed a normal distribution, while the median with interquartile range (IQR) was used for skewed variables. Group comparisons were performed using the t test or the Mann‒Whitney U test. Qualitative data are summarized as percentages and were compared using the chi-square test. Logistic regression analysis was performed to assess the association between RMS and CVD risk, including unadjusted and adjusted models (Model 1 was adapted for education, marital status, and sex; Model 2 added hypertension, dyslipidemia, diabetes, liver disease, kidney disease, smoking status, and alcohol status to Model 1; Model 3 further added age, SBP, DBP, pulse, TC, TG, HDL-C, LDL-C, CRP, and HbA1c to Model 2). Additionally, we employed restricted cubic splines (RCS), selecting four nodes representing the 5th, 35th, 65th, and 95th percentiles, to examine the relationship between RMS and CVD incidence. Subgroup analysis was also conducted by categorizing participants into different groups based on age (< 60 vs ≥ 60), sex, level of education, marital status, alcohol intake, hypertension, dyslipidemia, diabetes, liver disease, and kidney disease. We employed R version 4.1.2 and SPSS 27.0.1.0 for conducting the statistical analysis, and differences were deemed statistically significant when P was < 0.05.

## Results

### Baseline characteristics of participants

In this study, a total of 8,733 subjects were included in the analysis, including 4,600 (52.67%) females and 4,133 (47.33%) males, with an average age of 59, 1,152 (13.19%) CVD patients and 7,581 (86.81%) non-CVD patients, as shown in Table 1. Overall, compared with non-CVD patients, CVD patients tended to be older, female, and have higher SBP, DBP, TG, CRP, HbA1c, and BMI, as well as lower levels of HDL-C and grip strength (p ≤ 0.001). CVD patients also had a greater incidence of hypertension, dyslipidemia, diabetes, liver disease, and kidney disease (p ≤ 0.001). It was worth noting that CVD patients exhibited significantly lower RMS than non-CVD patients (P ≤ 0.001). Moreover, the baseline characteristics of the participants were analysed according to the quartiles of RMS (Table S1). A greater RMS was found to be associated with younger age, female sex, married, and lower education level (*P* < 0.001). As the RMS increased, there was a concurrent decrease in the incidence of CVD, hypertension, dyslipidemia, diabetes, and SBP, as well as in TG and CRP levels; meanwhile, grip strength and HDL-C increased. Furthermore, the baseline characteristics of the participants were also analysed based on the quartiles of grip strength. Greater grip strength was observed among individuals who were younger, male, married and had a lower education level (*P* < 0.001). With increasing grip strength, there was a decrease in the incidence rate of CVD along with diabetes occurrence as well as TC, HDL-C, and LDL-C levels; however, smoking and drinking behaviors increased, accompanied by elevated DBP, and ASM (Table S2).

**Table 1:** Characteristics of individuals included in the study

Variables	Total (*n* = 8,733)	With CVD (*n* = 1,152)	Without CVD (*n* = 7,581)	P
Age, median (IQR), years	59.00 (52.00, 65.00)	62.00 (56.00, 69.00)	58.00 (52.00, 65.00)	< 0.001
Sex, n (%)				
Female	4,600 (52.67%)	670 (58.16%)	3,930 (51.84%)	< 0.001
Male	4,133 (47.33%)	482 (41.84%)	3,651 (48.16%)	
Marital status, n (%)				< 0.001
Married	7,729 (88.50%)	972 (84.38%)	6,757 (89.13%)	
Others	1,004 (11.50%)	180 (15.63%)	824 (10.87%)	
Education level, n (%)				0.099
Elementary school or below	6,130 (70.19%)	823 (71.44%)	5,307 (70.00%)	
Middle school	2,490 (28.51%)	308 (26.74%)	2,182 (28.78%)	
College or above	113 (1.29%)	21 (1.82%)	92 (1.21%)	
SBP, median (IQR), years	127.67 (115.00, 143.00)	132.33 (118.00, 147.25)	127.00 (114.33, 142.33)	< 0.001
DBP, median (IQR), years	75.00 (67.33, 83.67)	76.33 (68.33, 85.33)	74.67 (67.33, 83.33)	< 0.001
Pulse, median (IQR), years	71.67 (65.33, 78.67)	72.00 (64.67, 79.33)	71.67 (65.33, 78.67)	0.565
Grip strength, median (IQR), kg	30.50 (24.25, 38.25)	28.50 (22.67, 35.39)	30.50 (24.50, 38.50)	< 0.001
Smoking status, n (%)				0.111
Yes	3,477 (39.81%)	434 (37.67%)	3,043 (40.14%)	
No	5,256 (60.19%)	718 (62.33%)	4,538 (59.86%)	
Drink status, n (%)				< 0.001
Yes	2,880 (32.98%)	281 (24.39%)	2,599 (34.28%)	
No	5,853 (67.02%)	871 (75.61%)	4,982 (65.72%)	
Hypertension, n (%)				< 0.001
Yes	2,176 (24.92%)	575 (49.91%)	1601 (21.12%)	
No	6,557 (75.08%)	577 (50.09%)	5,980 (78.88%)	
Dyslipidemia, n (%)				< 0.001
Yes	814 (9.32%)	274 (23.78%)	540 (7.12%)	
No	7,919 (90.68%)	878 (76.22%)	7,041 (92.88%)	
Diabetes, n (%)				< 0.001
Yes	502 (5.75%)	145 (12.59%)	357 (4.71%%)	
No	8,231 (94.25%)	1007 (87.41%)	7224 (95.29%)	
Liver disease, n (%)				< 0.001
Yes	351 (4.02%)	81 (7.03%)	270 (3.56%)	
No	8,382 (95.98%)	1071 (92.97%)	7,311 (96.44%)	
Kidney disease, n (%)				< 0.001
Yes	587 (6.72%)	142 (12.33%)	445 (5.87%)	
No	8,146 (93.28%)	1,010 (87.67%)	7,136 (94.13%)	
TC, median (IQR), mg/dL	190.59 (167.01, 215.72)	191.37 (168.17, 216.50)	190.59 (167.01, 215.34)	0.379
TG, median (IQR), mg/dL	105.32 (74.34, 154.88)	115.05 (80.54, 169.04)	103.54 (74.34, 152.22)	< 0.001
HDL-C, median (IQR), mg/dL	49.48 (40.40, 59.92)	46.97 (37.98, 57.99)	49.48 (40.98, 60.31)	< 0.001
LDL-C, median (IQR), mg/dL	114.05 (93.17, 137.63)	116.37 (93.94, 138.40)	114.05 (93.17, 137.24)	0.221
CRP, median (IQR), mg/L	1.04 (0.55, 2.17)	1.21 (0.66, 2.53)	1.01 (0.54, 2.12)	< 0.001
HbA1c, median (IQR), %	5.10 (4.90, 5.40)	5.20 (4.90, 5.50)	5.10 (4.90, 5.40)	< 0.001
BMI, median (IQR), kg/m^2^	23.14 (20.85, 25.79)	24.20 (21.53, 26.97)	23.02 (20.78, 25.58)	< 0.001
ASM, median (IQR)	17.64 (14.77, 20.42)	17.60 (14.99, 20.69)	17.65 (14.73, 20.38)	0.339
RMS, median (IQR)	1.80 (1.48, 2.11)	1.68 (1.36, 1.99)	1.82 (1.50, 2.12)	< 0.001

*Abbreviations*: *CVD *cardiovascular disease, *IQR *Interquartile range, *SBP *Systolic blood pressure, *DBP *Diastolic blood pressure, *TC *Total cholesterol, *TG *Triglyceride, *HDL-C *High-density lipoprotein cholesterol, *LDL-C *Low-density lipoprotein cholesterol, *CRP *C-reactive protein, *HbA1c *Glycated haemoglobin, *BMI *Body mass index, *ASM *Appendicular skeletal muscle mass, *RMS *Relative muscle strength

### Relationship between RMS and CVD incidence

**Fig. 2:**
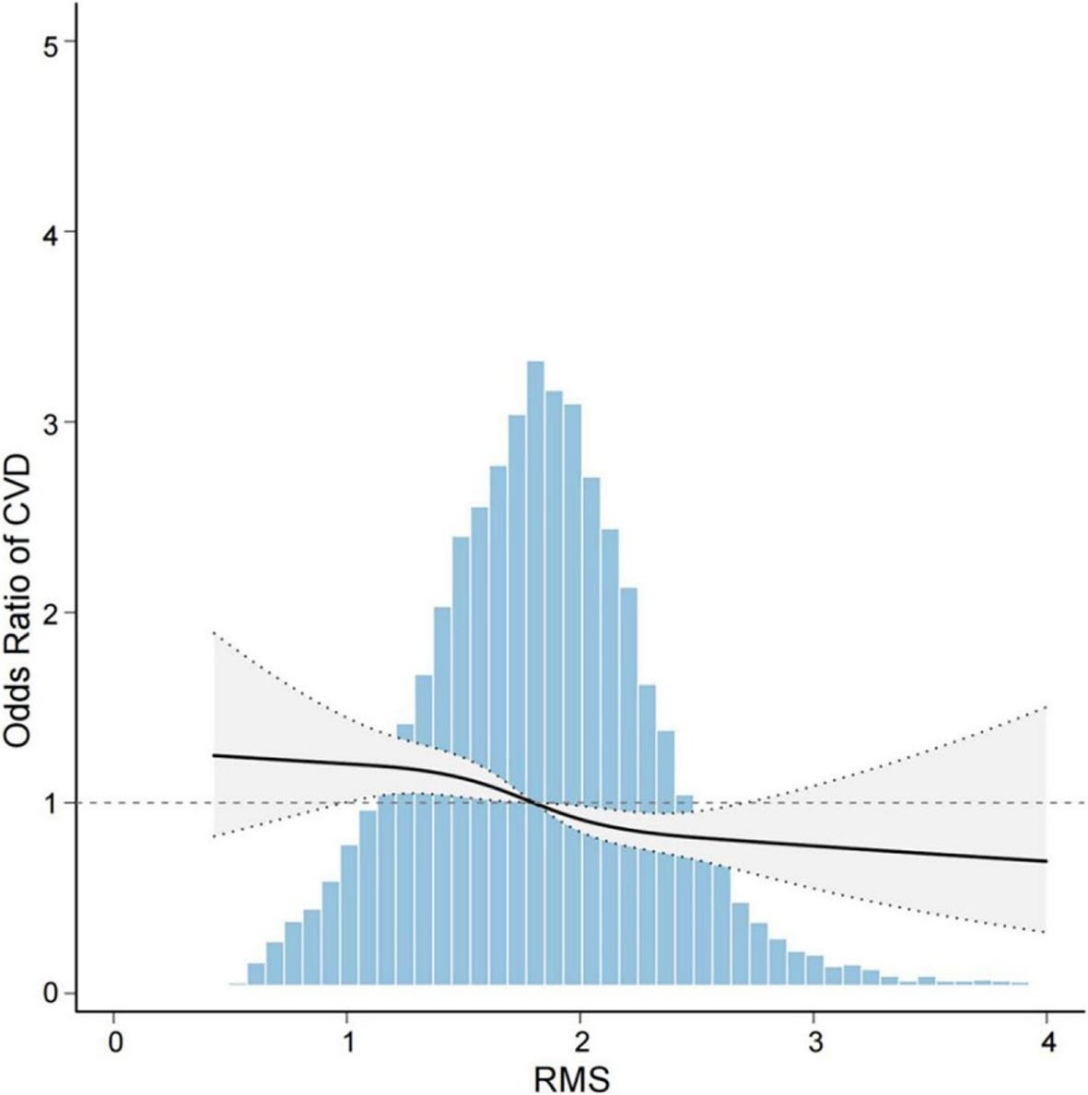
Dose‒response relationship between RMS and CVD incidence (P for nonlinearity = 0.555)

Furthermore, we performed multivariate logistic regression according to the quartile of RMS. According to the unadjusted analysis, there was a considerable inverse relationship between RMS and CVD (trend *P* < 0.001) (Table 2). The probability of CVD was reduced by 54% in Q4 compared to Q1 with a OR (95% CI) of (0.46 (0.38, 0.55)) in Q4. After adjusting for potential confounding factors, the effect of RMS on CVD incidence was still significant (trend *P* < 0.001). When adjusting models 1, 2, and 3, respectively, the CVD probability in Q4 is still significantly lower than in Q1, with a OR (95% CI) of (0.48 (0.40, 0.58)) in Model 1, (0.59 (0.49, 0.72)) in Model 2, and (0.70 (0.57, 0.85)) in Model 3. To further validate these findings from logistic regression analysis, RCS analysis was utilized to probe the relationship between RMS and CVD incidence. After controlling for all confounding factors (model 3), the RCS results demonstrated a consistent negative association between RMS and CVD (P for nonlinearity = 0.555) (Fig. 2). Additionally, a multivariate logistic regression analysis was conducted based on the quartile of grip strength. After not adjusting for confounders, the CVD probability in Q4 was reduced by 50% compared to that in Q1, with a OR (95% CI) of (0.50 (0.42, 0.61)) in Q4. Nevertheless, even after taking into account potential confounding factors, the correlation between grip strength and the probability of developing CVD remained statistically significant, with a OR (95% CI) of (0.49 (0.39, 0.62) in Model 1, 0.54 (0.42, 0.68) in Model 2, and 0.71 (0.55, 0.91)) in Model 3 (Table S3).

**Table 2:** Association between RMS and CVD in different models

Variable	N	Crude OR (95%CI)	P value	Model 1 OR (95%CI)	P value	Model 2 OR (95%CI)	P value	Model 3 OR (95%CI)	P value
RMS									
Q1	2,178	1(Ref)		1(Ref)		1(Ref)		1(Ref)	
Q2	2,175	0.79 (0.67, 0.93)	0.004	0.83 (0.72, 0.98)	0.023	0.92 (0.77, 1.09)	0.342	0.99 (0.83, 1.17)	0.870
Q3	2,222	0.57 (0.48, 0.68)	< 0.001	0.61 (0.51, 0.73)	< 0.001	0.72 (0.60, 0.87)	< 0.001	0.81 (0.67, 0.98)	0.027
Q4	2,158	0.46 (0.38, 0.55)	< 0.001	0.48 (0.40, 0.58)	< 0.001	0.59 (0.49, 0.72)	< 0.001	0.70 (0.57, 0.85)	< 0.001
Trend P		< 0.001		< 0.001		< 0.001		< 0.001	

*Abbreviations*: *Q1 to Q4 *quintile 1 to 4, *OR *odds ratio, *CI *confidence interval, *Ref *reference

Crude: unadjusted model

Model 1: adjusted for education + marital status + sex

Model 2: adjusted for Model 1 + hypertension + diabetes + dyslipidemia + liver disease + kidney disease + smoking status + drinking status

Model 3: adjusted for Model 2 + Age + SBP + DBP + Pulse + TC + TG + HDL-C + LDL-C + CRP + HbA1c

In addition we have conducted a sensitivity analysis which has confirmed the reliability and stability of the results (Table S4). In the sensitivity analysis we considered the sample weights, and the weighted results showed that RMS was still significantly correlated with CVD (*p* < 0.001), which was consistent with the above research results.

### Subgroup analyses

In addition, the present study conducted subgroup analyses considering factors such as age (< 60 vs ≥ 60), sex, education level, marital status, drink status, the presence of hypertension, diabetes, dyslipidemia, liver disease, and kidney disease to explore the correlation between RMS and CVD incidence. Subgroup analysis revealed an interaction effect between sex and RMS with CVD (p for interaction = 0.046). Compared with female sex, male sex had a more significant effect on reducing CVD probability with a OR (95% CI) of (0.68 (0.52, 0.90)) vs. 0.79 (0.68, 0.93)). Otherwise, no significant interaction was observed among the other subgroup variables (P for interaction > 0.05) in relation to the association between RMS and CVD (Table 3).

**Table 3:** Association between RMS and CVD incidence in different subgroups

Subgroup	Event (%)	OR (95%CI)	P value	P for interaction
Education level				
Elementary school or below	823 (13.43%)	0.73 (0.62, 0.85)	< 0.001	0.989
Middle school	308 (12.37%)	0.84 (0.64, 1.09)	0.183	
College or above	21 (18.58%)	0.72 (0.12, 4.12)	0.710	
Marital status				
Married	972 (12.58%)	0.75 (0.65, 0.88)	< 0.001	
Others	180 (17.93%)	0.74 (0.52, 1.04)	0.083	0.552
Sex				
Female	670 (14.57%)	0.79 (0.68, 0.93)	0.004	0.046
Male	482 (11.66%)	0.68 (0.52, 0.90)	0.006	
Age				
< 60	454 (9.66%)	0.70 (0.57, 0.86)	0.001	0.294
≥ 60	698 (17.32%)	0.75 (0.62, 0.90)	0.002	
Hypertension				
Yes	575 (26.42%)	0.78 (0.64, 0.96)	0.021	
No	577 (8.80%)	0.76 (0.63, 0.91)	0.003	0.571
Dyslipidemia				
Yes	274 (33.66%)	0.88 (0.64, 1.21)	0.433	
No	878 (11.09%)	0.73 (0.63, 0.85)	< 0.001	0.616
Diabetes				
Yes	145 (28.88%)	0.85 (0.54, 1.32)	0.471	
No	1007 (12.23%)	0.74 (0.64, 0.85)	< 0.001	0.414
Liver disease				
Yes	81 (23.08%)	0.89 (0.50, 1.60)	0.699	
No	1071 (12.78%)	0.75 (0.65, 0.86)	< 0.001	0.833
Kidney disease				
Yes	142 (24.19%)	0.59 (0.39, 0.89)	0.013	
No	1010 (12.40%)	0.78 (0.67, 0.91)	0.001	0.244
Drink status				
Yes	281 (9.76%)	0.76 (0.55, 1.04)	0.082	
No	871 (14.88%)	0.75 (0.64, 0.88)	< 0.001	0.753

## Discussion

In this cross-sectional study, our findings demonstrated a negative correlation between RMS and the likelihood of developing CVD among middle-aged and older individuals in China. After controlling for potential confounding variables, the multivariate logistic regression analysis indicated that elevated RMS levels were still linked to a decreased likelihood of developing CVD. Furthermore, the outcomes of the RCS analysis and subgroup examinations supported the link between RMS and CVD. According to the stratified analysis based on sex, the effect of RMS on reducing CVD probability was more significant in men than in women. CVD remains a prevalent global health issue that significantly increases the burden on healthcare systems worldwide. Therefore, early screening and prevention of CVD are particularly important. Our findings suggest that incorporating the assessment of RMS in early CVD screening and clinical practice may have a beneficial impact on the early prevention and diagnosis of CVD, ultimately reducing its incidence.

However, there is limited research on the correlation between RMS and CVD, a multitude of studies have investigated the linkages between sarcopenia-related characteristics (muscle strength and muscle mass) and CVD [23–25]. Many studies have utilized grip strength as a proxy for muscle strength [26]. Grip strength serves as a straightforward, rapid, reliable, and economical method for assessing muscle strength [15], which also correlates well with measurements obtained from other body parts, such as arms, legs, and trunks [27]. Compelling evidence suggests that grip strength is linked to CVD incidence. A systematic review encompassing more than 3.1 million individuals from more than 40 countries revealed that lower grip strength was associated with a greater probability of CVD-related mortality [28]. Additionally, other studies have indicated that grip strength could independently predict the prognosis of diabetic CVD patients [29]. Another study explored the correlation between muscle mass, muscle strength, and left ventricular function after excluding confounding factors such as coronary heart disease (CHD) and stroke, which may influence sarcopenia and CVD outcomes. Their findings demonstrated that higher grip strength was independently linked with improved left ventricular diastolic function irrespective of muscle mass variations [30]. It has been reported that a decrease in muscular strength occurs before a decrease in muscular mass among individuals as they age [31]. Furthermore, studies have shown that muscle strength decreases three times faster than muscle mass [32].

However, a bidirectional Mendelian randomization study investigating the relationship between sarcopenia syndrome and CVD suggested that appendicular lean mass serves as a protective factor against CHD, stroke, and myocardial infarction (MI). It was also noted that grip strength demonstrated a protective effect against CHD and MI, while no significant association with stroke was found [14]. In contrast, our study demonstrated an inverse correlation between grip strength and CVD, while no statistically significant association was found between muscle mass and CVD (*p* = 0.339). Hence, the associations between muscle strength, muscle mass, and CVD remain inconclusive.

Muscle quality is a relatively recent term for muscle strength related to muscle mass [33]. One study demonstrated that diabetes did not decrease muscle quality among elderly males; nevertheless, when the HbA1c level was ≥ 8.5%, the muscle quality of the lower limbs was significantly reduced [34]. Another study of the muscle quality index (MQI) and CVD incidence in the American population showed an inverse association between the MQI and CVD incidence (0.47 (0.35, 0.63), *P* < 0.001) in Model 1 without adjustment for covariates. However, in Model 4, adjusted for all variables, the MQI was not independently associated with CVD (0.79 (0.49, 1.29), *P* = 0.31) [35]. In contrast, our study concluded that a stable relationship remained after adjusting for all variables (trend *P* < 0.001), and the RCS curve and subgroup analysis further verified the stability of the two variables. These discrepancies may be due to disparities in age and race among the participants. Previous studies have shown differences in grip strength and body composition according to sex and race [36, 37], and men exhibit greater strength and muscle mass than women [38]. Our subgroup analysis showed that higher RMS in men was more significant in reducing CVD probability than in women. In addition, there are many ways to measure muscle quality [33]. Calculating upper limb muscle quality involves determining the ratio between grip strength and arm lean mass [39], and lower limb muscle quality is determined by calculating the ratio between quadriceps strength and leg lean mass [40]. Alternatively, an alternative method for evaluating muscle quality is to divide ASM by muscle strength [16], which was the approach taken in our study.

However, the precise mechanisms responsible for the correlation between RMS and CVD have yet to be fully elucidated. Recent research findings indicate that individuals suffering from chronic inflammatory conditions experience a faster decrease in muscle mass and strength than healthy individuals [41]. Inflammation can induce muscle atrophy by inhibiting muscle fibers [42], while damage to muscle strength and muscle mass further exacerbates inflammation [43]. Interleukin-6 (IL-6), tumor necrosis factor-α (TNF-α), and CRP have been reported as serum inflammatory markers for heart failure [44]. Inflammation, characterized by elevated levels of blood inflammatory markers, is a significant contributor to chronic disease, frailty, and premature mortality [45]. Moreover, the elevated levels of IL-6, TNF-α, and CRP are associated with decreased muscle mass and strength in older adults [46]. Our study also revealed that CRP levels increased with decreasing RMS. Therefore, it is plausible that the association between RMS and CVD stems from the reduction in RMS, which subsequently exacerbates the inflammatory response and elevates the levels of inflammatory indicators in the bloodstream, such as IL-6, TNF-α, and CRP, thereby augmenting the probability of developing CVD.

In conclusion, this study preliminarily investigated the correlation between RMS and the incidence of CVD, providing a simple calculation method for the early identification and prevention of CVD in clinical practice. However, this study also has some limitations that could be improved. First, it should be noted that this study was a cross-sectional study, which can only confirm the correlation between the RMS and the probability of developing CVD, but it cannot yet infer a causal relationship, and further cohort studies are needed to prove it. Second, this study focused solely on the Chinese population, especially regarding muscle mass. The utilization of an ASM formula based on the Chinese population necessitates caution when extrapolating these findings to other populations. Moreover, the identification of CVD in this research relied exclusively on participants' self-reported physician diagnostic data and lacked objective diagnostic criteria, potentially introducing reporting bias. In addition, we used the 2011 CHARLS data, which has a large sample size and is nationally representative, however, this data is older and may have some implications for the extrapolation of the study results. Finally, although we adjusted for as many factors as possible, we cannot exclude the mixed effects of some unknown factors.

## Conclusion

This study concluded that RMS is inversely associated with CVD, and the relationship is more pronounced in men. The RMS is a relatively easily obtained metric that facilitates clinical application. Therefore, we believe that RMS can contribute to the early identification and prevention of CVD.

### Supplementary Information

Supplementary Material 1. Table S1. Population characteristics by the quartiles of RMS. Table S2. Population characteristics by the quartiles of grip strength. Table S3. Associations between grip strength and CVD probability according to different models. Table S4. The correlation between RMS and CVD after increasing sample weights.

## Data Availability

The data that support the findings of this study are openly available in the CHARLS. (https://charls.charlsdata.com/pages/Data/2011-charls-wave1/zh-cn.html). And the derived data that were generated in the current study are available from the corresponding author upon reasonable request.

## Abbreviations

CVD: Cardiovascular disease
RMS: Relative muscle strength
CHARLS: China Health and Retirement Longitudinal Study
ASM: Appendicular skeletal muscle mass
RCS: Restricted cubic spline
SBP: Systolic blood pressure
DBP: Diastolic blood pressure
TC: Total cholesterol
HDL-C: High-density lipoprotein cholesterol
LDL-C: Low-density lipoprotein cholesterol
TG: Triglyceride
CRP: C-reactive protein
HbA1c: Glycated hemoglobin
SD: Standard deviatio
IQR: Interquartile range
OR: Odds ratio
CI: Confidence interval
Ref: Reference
Q1 to Q4: Quartiles 1 to 4
CHD: Coronary heart disease
MI: Myocardial infarction
MQI: Muscle quality index
IL-6: Interleukin-6
TNF-α: Tumor necrosis factor-α
