# Supplementary material for: Association between relative muscle strength and cardiovascular disease among middle-aged and older adults in China
Source: BMC Public Health. 2024 Jul 18;24:1928. doi: 10.1186/s12889-024-19473-y (PMC11256373; doi:10.1186/s12889-024-19473-y)
Supplement: Supplementary file 1 — Supplementary Material 1. Table S1. Population characteristics by the quartiles of RMS. Table S2. Population characteristics by the quartiles of grip strength. Table S3. Associations between grip strength and CVD probability according to different models. Table S4. The correlation between RMS and CVD after increasing sample weights. [file 12889_2024_19473_MOESM1_ESM.pdf]

Table S1. Population characteristics by the quartiles of RMS.

Variables	Total (n = 8733)	Q1 (n = 2178)	Q2 (n = 2175)	Q3 (n = 2222)	Q4 (n = 2158)	P
Age, median (IQR), years	59.00 (52.00, 65.00)	62.00 (56.00, 70.00)	59.00 (53.00, 66.00)	58.00 (52.00, 64.00)	55.00 (49.00, 61.00)	＜ 0.001
Sex, n (%)						
Female	4600 (52.67%)	1489 (68.37%)	1072 (49.29%)	898 (40.41%)	1141 (52.87%)	＜ 0.001
Male	4133 (47.33%)	689 (31.63%)	1103 (50.71%)	1324 (59.59%)	1017 (47.13%)	
Marital status, *n* (%)						＜ 0.001
Married,n(%)	7729 (88.50%)	1794 (82.37%)	1946 (89.47%)	2010 (90.46%)	1979 (91.71%)	
Others,n(%)	1004 (11.50%)	384 (17.63%)	229 (10.53%)	212 (9.54%)	179 (8.29%)	
Education level, n (%)						＜ 0.001
Elementary school or below,n(%)	6130 (70.19%)	1770 (81.27%)	1558 (71.63%)	1448 (65.17%)	1354 (62.74%)	
Middle school,n (%)	2490 (28.51%)	393 (18.04%)	586 (26.94%)	739 (33.26%)	772 (35.77%)	
College or above,n (%)	113 (1.29%)	15 (0.69%)	31 (1.43%)	35 (1.58%)	32 (1.48%)	
SBP, median (IQR), years	127.67 (115.00, 143.00)	131.00 (116.00, 148.00)	128.33 (115.67, 143.67)	127.00 (115.00, 141.67)	125.00 (113.25, 139.33)	＜ 0.001
DBP, median (IQR), years	75.00 (67.33, 83.67)	74.67 (67.33, 83.67)	75.00 (67.33, 83.67)	75.00 (67.33, 83.67)	74.67 (67.33, 83.33)	0.828
Pulse, median (IQR), years	71.67 (65.33, 78.67)	72.00 (65.67, 78.75)	71.33 (64.67, 78.33)	71. 17 (64.67, 78.33)	72.00 (64.67, 78.67)	0.025
Grip strength, median (IQR), kg	30.50 (24.25, 38.25)	21.20 (17.50, 25.00)	29.50 (25.00, 34.50)	35.00 (29.50, 40.50)	38.50 (31.05, 46.25)	＜ 0.001
Smoking status, n (%)						＜ 0.001
Yes	3477 (39.81%)	617 (28.33%)	885 (40.69%)	1080 (48.60%)	895 (41.47%)	
No	5256 (60.19%)	1561 (71.67%)	1290 (59.31%)	1142 (51.40%)	1263 (58.53%)	
Drink status, *n* (%)						＜0.001
Yes	2880 (32.98%)	448 (20.57%)	752 (34.57%)	898 (40.41%)	782 (36.24%)	
No	5853 (67.02%)	1730 (79.43%)	1423 (65.43%)	1324 (59.59%)	1376 (63.76%)	
CVD, n (%)						＜0.001
Yes	1152 (13.19%)	390 (17.91%)	319 (14.67%)	247 (11.12%)	196 (9.08%)	
No	7581 (86.81%)	1788 (82.09%)	1856 (85.33%)	1975 (88.88%)	1962 (90.92%)	
Hypertension, n (%)						＜ 0.001
Yes	2176 (24.92%)	696 (31.96%)	594 (27.31%)	487 (21.92%)	399 (18.49%)	
No	6557 (75.08%)	1482 (68.04%)	1581 (72.69%)	1735 (78.08%)	1759 (81.51%)	
Dyslipidemia, n (%)						＜ 0.001
Yes	814 (9.32%)	248 (11.39%)	207 (9.52%)	201 (9.05%)	158 (7.32%)	
No	7919 (90.68%)	1930 (88.61%)	1968 (90.48%)	2021 (90.95%)	2000 (92.68%)	
Diabetes, n (%)						＜ 0.001
Yes	502 (5.75%)	172 (7.90%)	127 (5.84%)	111 (5.00%)	92 (4.26%)	
No	8231 (94.25%)	2006 (92. 10%)	2048 (94.16%)	2111 (95.00%)	2066 (95.74%)	
Liver disease, n(%)						0.791
Yes	351 (4.02%)	92 (4.22%)	88 (4.05%)	92 (4.14%)	79 (3.66%)	
No	8382 (95.98%)	2086 (95.78%)	2087 (95.95%)	2130 (95.86%)	2079 (96.34%)	
Kidney disease, n (%)						0.032
Yes	587 (6.72%)	165 (7.58%)	148 (6.80%)	121 (5.45%)	153 (7.09%)	＜ 0.001
No	8146 (93.28%)	2013 (92.42%)	2027 (93.20%)	2101 (94.55%)	2005 (92.91%)	
TC, median (IQR), mg/dL	190.59 (167.01, 215.72)	192.72 (168.17, 219.20)	189.05 (166.62, 214.56)	190.59 (167.01, 214.56)	189.82 (166.62, 215.72)	0.015
TG, median (IQR), mg/dL	105.32 (74.34, 154.88)	109.30 (77.88, 159.30)	106.20 (75.22, 153.99)	103.54 (74.34, 152.22)	100.89 (70.80, 151.34)	＜ 0.001

HDL-C, median (IQR), mg/dL	49.48 (40.40, 59.92)	48.71 (39.82, 58.76)	48.71 (39.82, 59.54)	49.48 (40.59, 60.70)	50.64 (41.37, 61.08)	< 0.001
LDL-C, median (IQR), mg/dL	114.05 (93.17, 137.63)	116.75 (95.49, 140.34)	113.27 (91.62, 136.86)	112.89 (92.30, 135.31)	113.66 (93.17, 136.47)	< 0.001
CRP, median (IQR), mg/L	1.04 (0.55, 2.17)	1.15 (0.59, 2.43)	1.05 (0.58, 2.25)	1.03 (0.56, 2.09)	0.91 (0.51, 1.89)	< 0.001
HbA1c, median (IQR), %	5.10 (4.90, 5.40)	5.20 (4.90, 5.50)	5.10 (4.90, 5.40)	5.10 (4.90, 5.40)	5.10 (4.90, 5.40)	< 0.001
BMI, median (IQR), kg/m^2	23.14 (20.85, 25.79)	23.31 (20.84, 26.17)	23.28 (21.04, 26.01)	23.08 (20.78, 25.74)	22.89 (20.73, 25.35)	< 0.001
ASM, median (IQR)	17.64 (14.77, 20.42)	17.73 (15.68, 20.11)	17.88 (15.39, 20.70)	18.17 (15.15, 20.87)	15.89 (12.60, 19.87)	< 0.001

Abbreviations: CVD, cardiovascular disease; IQR, Interquartile range;Q1 to Q4, quintile 1 to 4； SBP, Systolic blood pressure; DBP, Diastolic blood pressure; TC, Total cholesterol; TG, Triglyceride; HDL-C, High-density lipoprotein cholesterol; LDL-C, Low-density lipoprotein cholesterol; CRP, C-reactive protein; HbA1c, Glycated haemoglobin. BMI, Body mass index; ASM, Appendicular skeletal muscle mass; RMS, Relative muscle strength.

Table S2. Population characteristics by the quartiles of grip strength.

Variables	Total (n = 8733)	Q1 (n = 2200)	Q2 (n = 2279)	Q3 (n = 2086)	Q4 (n = 2168)	P
Age, median (IQR), years	59.00 (52.00, 65.00)	63.00 (57.00, 70.00)	58.00 (52.00, 65.00)	58 (52.00, 64.00)	55.00 (49.00, 61.00)	< 0.001
Sex, n (%)						< 0.001
Female	4600 (52.67%)	1893 (86.05%)	1673 (73.41%)	850 (40.75%)	184 (8.49%)	
Male	4133 (47.33%)	307 (13.95%)	606 (26.59%）	1236 (59.25%)	1884 (91.51%)	
Marital status, n (%)						< 0.001
Married	7729 (88.50%)	1788 (81.27%)	1933 (87.45%)	1896 (90.89%)	2052 (94.65%)	
Others	1004 (11.50%)	412 (18.73%)	286 (12.55%)	190 (9.11%)	116 (5.35%)	
Education level, n (%)						< 0.001
Elementary school or below	6130 (70.19%)	1894 (86.09%)	1761 (77.27%)	1394 (66.83%)	1081 (49.86%)	
Middle school	2490 (28.51%)	301 (13.68%)	499 (21.90%)	657 (31.50%)	1033 (47.65%)	
College or above	113 (1.29%)	5 (0.23%)	19 (0.83%)	35 (1.68%)	54 (2.49%)	
SBP(IQR)	127.67 (115.00, 143.00)	129.83 (115.33, 146.67)	127.33 (114.67, 143.67)	126.67 (114.33, 142.33)	127.00 (115.67, 139.67)	< 0.001
DBP(IQR)	75.00 (67.33, 83.67)	74.00 (66.33, 82.00)	74.67 (66.67, 83.00)	75.00 (67.33, 84.00)	76.33 (69.00, 84.67)	< 0.001
Pulse(IQR)	71.67 (65.33, 78.67)	72.33 (65.67, 79.33)	71.00 (64.67, 78.00)	71.67 (65.00, 78.33)	71.67 (65.33, 78.67)	< 0.001
Smoking status, n (%)						< 0.001
Yes	3477 (39.81%)	387 (17.59%)	561 (24.62%)	991 (47.51%)	1538 (70.94%)	
No	5256 (60.19%)	1813 (82.41%)	1718 (75.38%)	1095 (52.49%)	630 (29.06%)	
Drink status, n (%)						< 0.001
Yes	2880 (32.98%)	359 (15.86%)	485 (21.28%)	766 (36.72%)	1280 (59.04%)	
No	5853 (67.02%)	1851 (84.14%)	1794 (78.72%)	1320 (63.28%)	888 (40.96%)	
CVD, n (%)						< 0.001
Yes	1152 (13.19%)	366 (16.64%)	316 (13.87%)	272 (13.04%)	198 (9.13%)	
No	7581 (86.81%)	1834 (83.36%)	1963 (86.13%)	1814 (86.96%)	1970 (90.87%)	
Hypertension, n (%)						< 0.001
Yes	2176 (24.92%)	638 (29.00%)	549 (24.09%)	517 (24.78%)	472 (21.77%)	
No	6557 (75.08%)	1562 (71.00%)	1730 (75.91%)	1569 (75.22%)	1696 (78.23%)	
Dyslipidemia, n (%)						0.651
Yes	814 (9.32%)	218 (9.91%)	205 (9.00%)	186 (8.92%)	205 (9.46%)	
No	7919 (90.68%)	1982 (90.09%)	2074 (91.00%)	1900 (91.08%)	1963 (90.54%)	
Diabetes, n (%)						0.007
Yes	502 (5.75%)	154 (7.00%)	137 (6.01%)	110 (5.27%)	101 (4.66%)	
No	8231 (94.25%)	2046 (93.00%)	2142 (93.99%)	1976 (94.73%)	2067 (95.34%)	

Liver disease, n (%)						0.343
Yes	351 (4.02%)	94 (4.27%)	93 (4.08%)	70 (3.36%)	94 (4.34%)	
No	8382 (95.98%)	2106 (95.73%)	2186 (95.92%)	2016 (96.64%)	2074 (95.66%)	
Kidney disease, n (%)						0.860
Yes	587 (6.72%)	144 (6.55%)	161 (7.06%)	135 (6.47%)	147 (6.78%)	
No	8146 (93.28%)	2056 (93.45%)	2118 (92.94%)	1951 (93.53%)	2021 (93.22%)	
TC Median (IQR), mg/dL	190.59 (167.01, 215.72)	194.85 (169.72, 220.75)	191.37 (168.94, 216.88)	188.27 (165.46, 212.63)	187. 11 (164.30, 212.24)	< 0.001
TG Median (IQR), mg/dL	105.32 (74.34, 154.88)	108.86 (77.88, 158.41)	106.20 (76.11, 153.10)	100.00 (71.68, 147.79)	103.54 (73.46, 158.41)	< 0.001
HDL-C, median (IQR), mg/dL	49.48 (40.40, 59.92)	50.64 (41.37, 60.70)	50.64 (41.75, 60.70)	48.71 (40.21, 59.54)	47.55 (38.66, 57.99)	< 0.001
LDL-C, median (IQR), mg/dL	114.05 (93.17, 137.63)	117.14 (94.72, 141.11)	114.82 (93.94, 138.79)	112.50 (91.24, 135.41)	112.50 (92.01, 133.76)	< 0.001
CRP, median (IQR), mg/dL	1.04 (0.55, 2.17)	1.06 (0.56, 2.32)	1.05 (0.57, 2.15)	1.03 (0.55, 2.17)	0.99 (0.54, 2.04)	0.110
HbA1c, median (IQR), mg/dL	5.10 (4.90, 5.40)	5.20 (4.90, 5.50)	5.10 (4.90, 5.40)	5.10 (4.90, 5.40)	5.10 (4.90, 5.40)	< 0.001
BMI, median (IQR), kg/m^2	23.14 (20.85, 25.79)	22.69 (20.34, 25.45)	23.22 (20.84, 25.94)	23.00 (20.69, 25.72)	23.61 (21.45, 25.96)	< 0.001
ASM, median (IQR)	17.64 (14.77, 20.42)	15.38 (13.44, 17.48)	15.90 (13.70, 18.11)	18.19 (15.86, 20.20)	21.22 (19.55, 23.01)	< 0.001
RMS, median (IQR)	1.80 (1.48, 2.11)	1.29 (1.07, 1.54)	1.73 (1.51, 2.01)	1.89 (1.71, 2.14)	2.11 (1.93, 2.32)	< 0.001

Abbreviations: CVD, cardiovascular disease; IQR, Interquartile range;Q1 to Q4, quintile 1 to 4; SBP, Systolic blood pressure; DBP, Diastolic blood pressure; TC, Total cholesterol; TG, Triglyceride; HDL-C, High-density lipoprotein cholesterol; LDL-C, Low-density lipoprotein cholesterol; CRP, C-reactive protein; HbA1c, Glycated haemoglobin. BMI, Body mass index; ASM, Appendicular skeletal muscle mass; RMS, Relative muscle strength.

Table S3. Association between grip strength and CVD in different models.

Variable	N	Crude OR (95%CI)	P value	Model 1 OR (95%CI)	P value	Model 2 OR (95%CI)	P value	Model 3 OR (95%CI)	P value
RMS									
Q1	2200	1(Ref)		1(Ref)		1(Ref)		1(Ref)	
Q2	2279	0.81 (0.69, 0.95)	0.01	0.81 (0.69, 0.96)	0.014	0.87 (0.73, 1.03)	0.108	0.96 (0.81, 1.15)	0.683
Q3	2086	0.75 (0.63, 0.89)	< 0.001	0.75 (0.62, 0.90)	< 0.002	0.80 (0.66, 0.97)	0.026	0.95 (0.77, 1.16)	0.584
Q4	2168	0.50 (0.42, 0.61)	< 0.001	0.49 (0.39, 0.62)	< 0.001	0.54 (0.42, 0.68)	< 0.001	0.71 (0.55, 0.91)	0.007
Trend P		< 0.001		< 0.001		< 0.001		0.024	

Abbreviations: Q1 to Q4, quintile 1 to 4; OR, odds ratio; CI, confidence interval; Ref, reference.
Crude: unadjusted model.
Model 1: adjusted for education + marital status + sex.
Model 2: adjusted for Model 1 + hypertension + diabetes + dyslipidemia + liver disease + kidney disease + smoking status + drinking status.
Model 3: adjusted for Model 2 + Age + SBP + DBP + Pulse + TC + TG + HDL-C + LDL-C + CRP + HbA1c.

Table S4. The correlation between RMS and CVD after increasing sample weights.

Variable	N	Crude OR (95%CI)	P value	Model 1 OR (95%CI)	P value	Model 2 OR (95%CI)	P value	Model 3 OR (95%CI)	P value
RMS									
Q1	2200	1(Ref)		1(Ref)		1(Ref)		1(Ref)	
Q2	2279	0.85 (0.65, 1.10)	0.214	0.92 (0.71, 1.20)	0.558	1.01 (0.79, 1.30)	0.943	1.07 (0.84, 1.37)	0.574
Q3	2086	0.57 (0.44, 0.73)	< 0.001	0.63 (0.49, 0.81)	< 0.001	0.73 (0.56, 0.94)	0.017	0.83 (0.63, 1.09)	0.179
Q4	2168	0.46 (0.36, 0.58)	< 0.001	0.49 (0.39, 0.63)	< 0.001	0.62 (0.49, 0.80)	< 0.001	0.76 (0.59, 0.98)	0.031
Trend P		< 0.001		< 0.001		< 0.001		<0.001	

Abbreviations: Q1 to Q4, quintile 1 to 4; OR, odds ratio; CI, confidence interval; Ref, reference.
In the model, we considered the sampling weights for complex sample designs.
Crude: unadjusted model.
Model 1: adjusted for education + marital status + sex.
Model 2: adjusted for Model 1 + hypertension + diabetes + dyslipidemia + liver disease + kidney disease + smoking status + drinking status.
Model 3: adjusted for Model 2 + Age + SBP + DBP + Pulse + TC + TG + HDL-C + LDL-C + CRP + HbA1c.
